# Socioeconomic status and intrinsic capacity trajectories among middle-aged and older adults in China: mediating role of cognitive leisure activities

**DOI:** 10.1016/j.jnha.2026.100809

**Published:** 2026-02-21

**Authors:** Mingyan Qing, Jiang lan Wang, Lu Wang, Linyan Xie, Yuqin He, Longyan Ran, Xiuhong Wang

**Affiliations:** a School of Nursing, Guizhou Medical University, Guiyang, Guizhou, China; b The Affiliated Hospital of Guizhou Medical University, Guiyang, Guizhou, China

**Keywords:** Intrinsic capacity, Socioeconomic status, Cognitive leisure, Middle-aged and older adults

## Abstract

**Objectives:**

This study aimed to examine the association between socioeconomic status (SES) and intrinsic capacity (IC) trajectories among Chinese adults aged ≥45 years and evaluate the mediating role of cognitive leisure activities (CLA) on this relationship.

**Design:**

A cohort study.

**Setting and participants:**

Data were obtained from the China Health and Retirement Longitudinal Study (CHARLS, 2011–2015). The analytic sample comprised 3,922 community-dwelling adults aged ≥45 years with complete data on SES, CLA, and five domains of IC.

**Measurements:**

IC trajectories were identified utilizing group-based trajectory modeling (GBTM) across locomotion, vitality, psychology, cognition, and sensory domains. SES was evaluated utilizing education and household wealth, whereas CLA was quantified by activity frequency. Multinomial logistic regression was employed to investigate the associations among SES, CLA, and IC trajectories, whereas mediation analysis was performed to evaluate the indirect effects of CLA.

**Results:**

Among the 3,922 participants, GBTM identified four distinct IC trajectories from 2011 to 2015: High-level stable (n = 2,620; 66.8%), moderate-level declining (n = 620; 15.8%), low-level improving (n = 476; 12.1%), and low-level declining (n = 206; 5.3%). After adjusting for covariates, higher SES was associated with lower odds of belonging to less favorable trajectories compared to the high-level stable group. The corresponding odds ratios (ORs) were 0.830 (95% confidence interval [CI]: 0.769–0.896), 0.943 (95% CI: 0.902–0.986), and 0.899 (95% CI: 0.855–0.945), respectively. Higher levels of CLA were similarly associated with reduced odds of these trajectories (ORs = 0.480, 0.696, and 0.631; all *p <* 0.001). Mediation analysis revealed a significant indirect effect of SES on IC through CLA (a × b = 0.004; 95% bootstrap CI: 0.003–0.007), whereas the direct effect remained significant (c′ = 0.037, *p <* 0.01), indicating partial mediation.

**Conclusion:**

Higher SES correlated with more favorable IC trajectories among Chinese adults aged ≥45 years, with CLA partially mediating this association and highlighting the significance of behavioral engagement in mitigating socioeconomic disparities in healthy aging.

## 1 Introduction

Intrinsic capacity (IC), introduced by the World Health Organization (WHO) in 2015, is defined as the composite of an individual’s physical, cognitive, psychological, and sensory abilities that collectively support functional health throughout the aging process [1,2]. Within the healthy aging framework, IC signifies a transition from disease-centered methodologies toward function-oriented evaluation and management. Longitudinal variations in IC have been associated with functional ability, quality of life, and adverse health outcomes, including disability, falls, and mortality among middle-aged and older adults [[3], [4], [5]]. Previous studies demonstrated that declines in IC are correlated with increased long-term mortality risk [6], reduced social participation [7], type 2 diabetes [8], and depressive symptoms [9]. Recent research has further highlighted the potentially bidirectional relationship between IC and functional ability [10] and has investigated factors influencing IC trajectories, including social determinants of health [11], internet use [12], and multicomponent interventions [13]. These findings have significant implications for formulating targeted health promotion strategies for middle-aged and older adults.

Socioeconomic status (SES) is widely recognized as a primary social factor of health and aging outcomes. SES, commonly measured through educational achievement and household wealth, influences access to healthcare, nutrition, living conditions, and environments conducive to cognitive engagement [9,14,15]. Middle-aged and older adults with limited socioeconomic resources encounter increased risks of multimorbidity, accelerated functional decline, and diminished cognitive reserve, which collectively may accelerate deterioration in IC [[16], [17], [18], [19]]. Although robust associations between SES and overall health outcomes are well established, the mechanisms linking socioeconomic disparities to longitudinal trajectories of IC remain unclear, especially in diverse cultural contexts such as China.

Emerging evidence indicates that cognitive leisure activities (CLAs)—including reading, strategic games (chess or cards), stock trading, and internet usage correlate with improved cognitive and psychological well-being, potentially aiding in IC preservation [20,21]. Engagement in CLAs may enhance cognitive reserve, alleviate psychological stress, and foster social interaction, with possibly greater advantages for socioeconomically disadvantaged middle-aged and older adults [[22], [23], [24]]. Accordingly, participation in CLA may represent an important behavioral pathway linking SES to IC, potentially alleviating socioeconomic differences in aging outcomes. Despite these theoretical connections, longitudinal evidence regarding the impact of SES on dynamic IC trajectories through participation in CLA remains limited. Most existing studies utilize cross-sectional designs, which are insufficient for capturing temporal variations in IC and individual heterogeneity. Consequently, heterogeneity in longitudinal IC trajectories remains insufficiently defined. Trajectory-based modeling provides a robust framework to encapsulate this heterogeneity and outline significant developmental patterns over time.

To address these gaps, we utilized longitudinal data from the China Health and Retirement Longitudinal Study (CHARLS) to investigate the correlation between SES and IC trajectories among middle-aged and older Chinese adults. Specifically, we evaluated the mediating role of cognitive leisure activities, thereby offering empirical evidence on modifiable behavioral pathways through which socioeconomic disparities influence IC in later life.

## 2 Materials and methods

### 2.1 CHARLS

CHARLS is a nationally representative longitudinal survey of Chinese adults aged ≥ 45 years. Initiated in 2011, CHARLS conducts biennial follow-up assessments utilizing face-to-face computer-assisted interviews. These assessments collect comprehensive information on demographic characteristics, family structure, health status and functioning, healthcare usage and insurance, employment and retirement, income and assets, housing conditions, and biomarker and blood measurements. Survey waves occurred in 2011, 2013, 2015, 2018, and 2020, encompassing 150 counties and 450 communities across 28 provinces in China. The survey includes approximately 19,000 respondents from 12,400 households. The CHARLS study protocol was approved by the Biomedical Ethics Review Board of Peking University (IRB No. IRB00001052-11015), covering the main household survey and associated anthropometric measurements.

### 2.2 Study sample

**Fig. 1:**
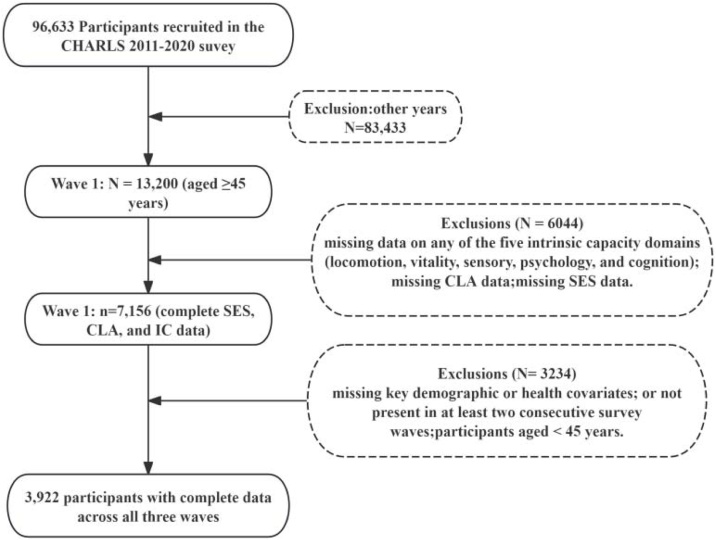
Participant selection process. **Note**: The y-axis represents Z-standardized intrinsic capacity (IC) scores. Shaded areas denote the 95% confidence intervals (CIs).

This longitudinal cohort study involved participants aged ≥45 years selected from the 2011 to 2015 waves of the CHARLS. Initially,39,600 respondents participated across the three survey waves. The 2018 and 2020 follow-up waves were excluded due to the absence of essential physical measurements required for constructing IC, specifically in the locomotion and vitality domains (short physical performance battery [SPPB], grip strength, and lung function). The variables were structurally absent and could not be imputed; hence, these waves were excluded from the analysis. All independent variables, including SES, CLA, and other covariates, were evaluated at the same three time points (2011, 2013, and 2015) as IC, ensuring temporal consistency among variables. Participants were excluded for missing data on IC, CLA, or essential covariates. After applying the exclusion criteria, a final sample of 3,922 participants was retained for analysis. IC was modeled longitudinally using group-based trajectory modeling (GBTM). The association between SES and IC trajectory membership was analyzed using multinomial logistic regression. Fig. 1 depicts the participant selection process.

### 2.3 IC

IC was operationalized following the WHO Integrated Care for Older People (ICOPE) guidelines and utilized scoring protocols described in previous studies, including the systematic review by Lopez-Ortiz et al. and cohort studies by Zhao et al. and Quan et al. [2,[25], [26], [27]]. The total IC score varied from 0 to 10, with each of the five IC domains (locomotion, vitality, psychological, cognition, and sensory) rated from 0 to 2. (1) **Locomotion**. Locomotion was evaluated using the SPPB (range 0–12), including a 4-m walking speed test, standing balance, and five repeated chair rises. The SPPB total score was classified into three levels and correlated with a 0–2 locomotion score (0–2, 3–9, and 10–12, respectively). (2) **Sensory**. Sensory capacity comprised vision and hearing. Vision included self-rated distance vision and near vision, and hearing was assessed by self-rated hearing. For each item, responses of excellent/very good/good were scored as 1, fair as 0.5, and poor as 0 (consistent with the prespecified scoring rules). The sensory domain score (0–2) was calculated by aggregating the three item scores based on prespecified criteria. (3) **Vitality**. Vitality comprised lung capacity and grip strength. Lung capacity was defined as the maximum of three spirometry measurements and dichotomized using sex-specific thresholds (350 L/min for men and 220 L/min for women). Grip strength was defined as the maximum dominant-hand value and dichotomized utilizing sex-specific thresholds (28 kg for men and 18 kg for women). The vitality domain score (0–2) was calculated by combining the two sub-dimensions. (4) **Psychology**. Psychological capacity was evaluated using the 10-item Centre for Epidemiological Studies Depression Scale (CESD-10; range 0–30). Scores were classified into three levels and mapped to a 0–2 psychological scores (20–30 = 0; 10–19 = 1; 0–9 = 2). (5) **Cognition**. Cognitive function in CHARLS was assessed using the Telephone Interview of Cognitive Status (TICS), adapted from the U.S. Health and Retirement Study (HRS). The assessment comprises two components: mental intactness and episodic memory. For each component, participants were assigned a score of 0 if their performance was more than one standard deviation below the sample mean, and 1 otherwise. The cognition score was calculated by summing the two component scores, yielding a total range of 0–2, with higher scores indicating better cognitive function.The comprehensive operationalization and scoring criteria (including item-level scoring and cut-off values for each domain) are provided in the Supplementary Material.

### 2.4 Socioeconomic Status

SES was evaluated utilizing two components: educational achievement and total household wealth. Educational attainment was categorized into three levels: primary or less than upper secondary education (score = 0), upper secondary or vocational education (score = 1), and tertiary education (score = 2). Total household wealth was defined as the sum of all household assets, including real estate, business holdings, vehicles, and savings accounts, excluding liabilities such as debts or loans. At each survey wave, total household wealth was categorized into quartiles, with scores ranging from 0 (lowest quartile) to 3 (highest quartile). The overall SES score was calculated by summing the scores for educational attainment and household wealth [14].

### 2.5 Cognitive leisure activities

Participation in CLA was treated as the independent variable in this study and was measured using data from the CHARLS. In the 2013 survey wave, participants were asked about their participation in various social activities during the preceding month. Activities classified as CLA included playing mahjong, chess, or cards; attending community clubs; stock investment; and internet use. Participation in each activity was classified as 0 (non-participation) or 1 (participation). The responses from the three activities were summed to create the Cognitive Leisure Activity Index (CLAI) [28]. Higher CLAI scores signify greater engagement in cognitive leisure activities.

### 2.6 Covariates

The analyses were adjusted for various sociodemographic and health-related covariates, including age (45–59, 60–69, 70–79, and ≥80 years), sex (female or male), education (below primary school, primary school, middle school, and high school or above), marital status (married or other), and type of residence (rural or urban). Health-related behaviors included smoking and alcohol consumption, both coded as binary variables (0 = no, 1 = yes).

### 2.7 Statistical analysis

Table 1 presents the baseline characteristics of the IC trajectory groups. Continuous variables (IC, SES, and CLA) are presented as means ± standard deviations, whereas categorical variables are reported as frequencies and percentages. Between-group differences were evaluated using chi-square tests for categorical variables and either analysis of variance or Kruskal–Wallis tests for continuous variables, as appropriate based on the Shapiro–Wilk test for normality.

**Table 1:** Baseline characteristics of participants across IC trajectory groups.

Variable	IC Trajectory Group				*P* value
	Moderate-to-high Stable Group (n = 2620)	Low-level Declining Group (n = 206)	Moderate-level Declining Group (n = 620)	Low-level Improving Group (n = 476)	
Age Group					
45–59	1846(70.46)	87(42.23)	355(57.26)	266(55.88)	
60–69	604(23.05)	86(41.75)	200(32.26)	158(33.19)	<0.001
70–79	164(6.26)	29(14.08)	64(10.32)	47(9.87)	
≥ 80	6(0.23)	4(1.94)	1(0.16)	5(1.05)	
Female, n (%)	1115(42.56)	109(52.91)	327(52.74)	256(53.78)	<0.001
Educational level, n (%)					
Below the primary school	594(22.67)	121(58.74)	259(41.77)	189(39.71)	<0.001
Primary school	710(27.10)	48(23.30)	179(28.87)	153(32.14)	
Lower secondary school	846(32.29)	30(14.56)	134(21.61)	99(20.80)	
Upper secondary school or above	470(17.94)	7(3.40)	48(7.74)	35(7.35)	
Married (versus other), n (%)	2451(93.55)	178(86.41)	555(89.52)	423(88.87)	<0.001
Urban residence(versus rural), n (%)	1564(59.69)	166(80.58)	437(70.48)	332(69.75)	<0.001
Number of chronic diseases					
0	1031(39.35)	30(14.56)	142(22.90)	99(20.80)	<0.001
1	799(30.50)	52(25.24)	179(28.87)	134(28.15)	
≥ 2	790(30.15)	124(60.19)	299(48.23)	243(51.05)	
Current Drinking, n (%)	1050(40.08)	61(29.61)	190(30.65)	154(32.35)	<0.001
Self-rated Health, n (%)					
Very good	245(9.35)	3(1.46)	17(2.74)	12(2.52)	<0.001
Good	583(22.25)	11(5.34)	82(13.23)	39(8.19)	
Fair	1462(55.80)	69(33.50)	322(51.94)	236(49.58)	
Poor	301(11.49)	99(48.06)	164(26.45)	173(36.34)	
Very poor	29(1.11)	24(11.65)	35(5.65)	16(3.36)	
SES, mean ± SD	2.49 ± 1.38	1.67 ± 1.34	2.01 ± 1.36	2.02 ± 1.33	<0.001
CLA, mean ± SD	0.29 ± 0.49	0.15 ± 0.35	0.19 ± 0.40	0.17 ± 0.37	<0.001
IC, mean ± SD	9.52 ± 0.61	5.94 ± 0.84	7.79 ± 0.69	6.35 ± 0.72	<0.001

**Note:** SES, socioeconomic status; CLA, cognitive leisure activities; IC, intrinsic capacit,SES, CLA, and IC were analyzed as continuous variables. IC total scores were standardized as Z-scores prior to trajectory modeling.

Before trajectory modeling, total IC scores were standardized as Z-scores utilizing the pooled mean and standard deviation from all survey waves (2011–2015), to ensure temporal comparability. GBTM was employed to identify heterogeneous IC trajectories from 2011 to 2015, utilizing five IC domains. Models delineating a number of trajectory classes were estimated and compared. Model selection was based on overall fit indices (AIC, BIC, and SABIC), classification quality metrics (entropy, AvePP, and OCC), and the proportion of participants across each class. Likelihood ratio tests, including the Lo–Mendell–Rubin test (LMRT) and bootstrap likelihood ratio test, were employed to assess improvements in model fit. The final model was selected by simultaneously evaluating model fit, classification accuracy, parsimony, interpretability, and avoidance of classes comprising <5% of the total sample.

Multinomial logistic regression was employed to investigate associations between IC trajectory membership and SES and CLA. Causal mediation analysis was performed to investigate if baseline CLA (2011) mediated the association between baseline SES (2011) and IC assessed at follow-up (2015). To establish temporal sequencing, SES, CLA, and all covariates were assessed at baseline, whereas IC measured in 2015 was regarded as the outcome. Raw IC scores were utilized in mediation analyses, whereas Z-standardized IC scores were utilized only for trajectory modeling. Indirect effects were estimated utilizing 2,000 bootstrap resamples to obtain 95% confidence intervals (CI). All analyses were conducted using RStudio and IBM Statistical Package for the Social Sciences Statistics (version 29.0). A two-sided *p* < 0.05 was considered statistically significant.

## 3 Results

### 3.1 Characteristics of participants by IC trajectory group

After applying the eligibility criteria, 3,922 participants from the CHARLS dataset (2011–2015) were included. Four IC trajectory groups were identified using GBTM: Moderate-to-high stable (n = 2,620), low-level declining (n = 206), moderate-level declining (n = 620), and low-level improving (n = 476) (Table 1). Baseline characteristics were derived from the 2011 wave. Significant differences among trajectory groups were observed in age, sex, educational level, marital status, residence, chronic disease burden, drinking status, self-rated health (SRH), and continuous measures of SES, CLA, and baseline IC (all *p <* 0.001). Compared with the moderate-to-high stable group, participants in the low-level declining group were older and exhibited a significantly higher prevalence of multimorbidity (≥2 chronic diseases: 60.19% versus 30.15%). Additionally, they reported significantly poorer self-rated health, with higher proportions rating their health as poor (48.06% versus 11.49%) or very poor (11.65% versus 1.11%). The low-level declining group exhibited the lowest mean scores for SES (1.67 ± 1.34), CLA (0.15 ± 0.35), and baseline IC (5.94 ± 0.84). Conversely, the moderate-to-high stable group exhibited the highest corresponding values (2.49 ± 1.38, 0.29 ± 0.49, and 9.52 ± 0.61, respectively). The moderate-level declining and low-level improving groups generally exhibited intermediate baseline profiles.

### 3.2 Trajectories of intrinsic capacity

**Fig. 2:**
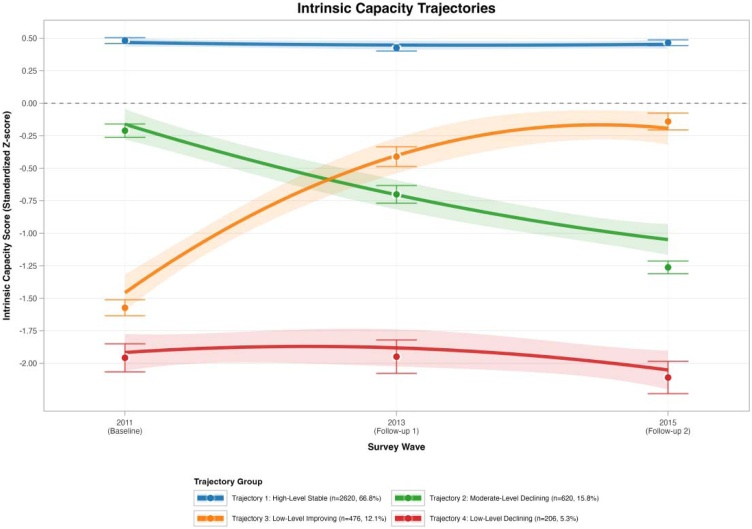
Trajectory of the IC.

Using GBTM with a quadratic (second-order) time function, four unique IC trajectories were identified between 2011 and 2015 (Fig. 2). The largest trajectory group was the high-level stable trajectory (Class 2; n = 2,620; 66.8%). Two trajectories exhibited declining patterns, namely the moderate-level declining trajectory (Class 4; n = 620; 15.8%) and the low-level declining trajectory (Class 1; n = 206; 5.3%). Conversely, one trajectory demonstrated an improving pattern, the low-level improving trajectory (Class 3; n = 476; 12.1%).

Table 2 presents the model fit for solutions including 1–6 classes. The AIC, BIC, and sample-size adjusted BIC (SABIC) decreased significantly as the number of classes increased from one to four, attaining their minimum values in the four-class solution (AIC = 30,047.53; BIC = 30,147.92; SABIC = 30,114.65), with successful model convergence. Adding extra classes did not enhance model fit; both the five- and six-class configurations yielded higher AIC, BIC, and SABIC values than the four-class solution, and the pLMRT was non-significant for both models (*p* = 1.000). Entropy values further corroborated the four-class solution (entropy = 0.787), whereas entropy decreased substantially in the five- and six-class models (0.538 and 0.452, respectively), signifying poorer classification due to over-extraction of classes. Classification diagnostics for the selected four-class model (Table 3) demonstrated satisfactory class differentiation, with class-specific average posterior probabilities (AvePP) of 0.935, 0.763, 0.734, and 0.875 (all ≥0.70) and corresponding odds of correct classification (OCC) values of 7.6, 15.8, 19.7, and 121.7. The smallest class represented 5.3% of the sample (n = 206) and demonstrated high classification certainty (AvePP = 0.875; OCC = 121.7).

**Table 2:** Model fit statistics for group-based trajectory models.

Classes	AIC	BIC	ΔBIC	SABIC	Ej	pLMRT	AvepP %	Proportions Per Class (%)
1	33394.46	33419.56	NA	NA	NA	NA	NA	100.0%
2	30870.36	30920.56	−2499.00	30903.92	0.814	<0.001	90.7	75.6%, 24.4%
3	30439.67	30514.96	−405.60	30490.01	0.795	<0.001	86.9	65.9%, 4.1%, 30.0%
4	30047.53	30147.92	−367.04	30114.65	0.787	<0.001	82.7	5.3%, 66.8%, 12.1%,15.8%
5	30055.53	30181.02	33.10	30139.43	0.538	1.000	71.0	63.1%, 0.0%, 12.1%, 19.5%, 5.3%
6	30063.53	30214.12	33.10	30164.21	0.452	1.000	68.5	12.1%, 62.9%, 19.7%, 5.3%, 0.0%, 0.0%

**Note**: Lower AIC/BIC/SABIC indicates a better fit. Entropy (Ej) closer to 1 indicates clearer classification. ΔBIC is the change in BIC compared with the (k−1)-class model. Proportions are illustrated in the model output order.

**Table 3:** Classification diagnostics for the selected 4-class model, by trajectory group.

Trajectory group	GBTM Class	N	AvePP (%)	OCC	P_j_ (%)	π_j_ (%)
Trajectory 1: High-level stable	Class 2	2620	93.5	7.6	66.8	65.3
Trajectory 2: Moderate-level declining	Class 4	620	76.3	15.8	15.8	16.9
Trajectory 3: Low-level improving	Class 3	476	73.4	19.7	12.1	12.3
Trajectory 4: Low-level declining	Class 1	206	87.5	121.7	5.3	5.4

**Note**: Pj is the assigned (modal) class proportion. πj is the estimated class proportion computed as the mean posterior probability. AvePP is the mean posterior probability for individuals assigned to each class. OCC is computed using the standard definition: OCCj = [AvePP_j/(1−AvePP_j)] / [πj/(1−πj)].

### 3.3 Socioeconomic Status and other predictors of IC trajectories

As illustrated in Table 4, multinomial logistic regression analyses demonstrated that higher engagement in CLA was consistently associated with lower odds of membership in less favorable IC (IC) trajectory groups, compared with the Moderate-to-High Stable group. Specifically, higher CLA scores were associated with reduced odds of belonging to the low-level declining group (odds ratio [OR] = 0.480, 95% CI: 0.373–0.617), the Moderate-Level Declining group (OR = 0.696, 95% CI: 0.615–0.788), and the low-level improving group (OR = 0.631, 95% CI: 0.548–0.727), with all associations reaching statistical significance (*p <* 0.001). Higher SES was likewise associated with a lower likelihood of unfavorable IC trajectory membership. Participants with higher SES had significantly reduced odds of being classified into the low-level declining (OR = 0.830, 95% CI: 0.769–0.896, *p <* 0.001), moderate-level declining (OR = 0.943, 95% CI: 0.902–0.986, *p <* 0.01), and low-level improving groups (OR = 0.899, 95% CI: 0.855–0.945, *p <* 0.001), relative to the Moderate-to-High Stable trajectory. With respect to effect modification, none of the interaction terms between SES and residential setting (urban versus rural) reached statistical significance across trajectory comparisons (all *p* > 0.05), indicating that the associations between SES and IC trajectory membership did not differ significantly by place of residence. All models were adjusted for age, sex, marital status, residence, educational level, chronic disease burden, smoking, and drinking.

**Table 4:** Multinomial logistic regression results for IC trajectory groups.

Variable	Low-level Declining Group		Moderate-level Declining Group		Low-level Improving Group	
	OR (95% CI)	*P* value	OR (95% CI)	*P* value	OR (95% CI)	*P* value
CLA Score	0.480 (0.373∼0.617)	<0.001	0.696 (0.615∼0.788)	<0.001	0.631 (0.548∼0.727)	<0.001
SES Score	0.830 (0.769∼0.896)	<0.001	0.943 (0.902∼0.986)	<0.01	0.899 (0.855∼0.945)	<0.001
SES × Residence	1.060 (0.901∼1.248)	0.482	0.926 (0.850∼1.009)	0.079	1.075 (0.978∼1.182)	0.132

**Note:** Odds ratios (ORs) and 95% confidence intervals (CIs) are reported. Only key predictors are presented. All models were adjusted for age, sex, marital status, residence (urban/rural), number of chronic diseases, current smoking, and current drinking. SES = socioeconomic status; CLA = cognitive leisure activities. The reference group was the moderate-to-high stable trajectory group.

### 3.4 Mediating role of CLA in the association between SES and IC

**Fig. 3:**
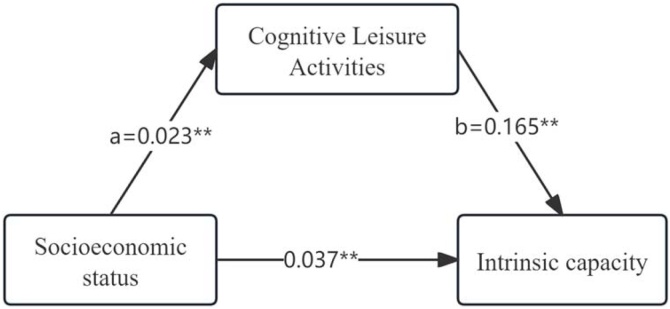
Mediation pathway diagram. Note: All coefficients are standardized. Bootstrap confidence intervals are percentile bootstrap; values are rounded to three decimals. Adjusted for age, gender, marital status, rural residence, educational level, number of chronic diseases, current smoking, and current drinking. *p < 0.05; **p < 0.01.

Mediation analysis was conducted to examine whether baseline CLA mediated the association between baseline SES and IC (IC) at follow-up in 2015 (Fig. 3). All coefficients were standardized. After adjustment for age, sex, marital status, residence, educational level, chronic disease burden, smoking, and drinking, SES exhibited a significant total effect on IC (c = 0.041, *p <* 0.01). When CLA was included as a mediator, the direct effect of SES on IC remained statistically significant (c′ = 0.037, *p <* 0.01). Additionally, SES was positively associated with CLA (a = 0.023, *p <* 0.01), and CLA was positively associated with IC (b = 0.165, *p <* 0.01). The indirect effect of SES on IC through CLA was statistically significant (a × b = 0.004; 95% bootstrap CI: 0.003–0.007), indicating partial mediation. The indirect pathway via CLA accounted for approximately 9.8% of the total effect of SES on IC.

## 4 Discussion

This study utilized longitudinal data from the CHARLS between 2011 and 2015 to delineate four distinct trajectories of IC among middle-aged and older adults: a moderate-to-high stable group (66.8%), a low-level declining group (5.3%), a moderate-level declining group (15.8%), and a low-level improving group (12.1%). Collectively, these trajectories demonstrate heterogeneous patterns of stability, decline, and improvement in IC over time, consistent with previous evidence highlighting substantial inter-individual variability in functional aging. Selecting the four-class solution was supported by model-fit indices and classification diagnostics and by the interpretability and adequate size of each trajectory group.

Regarding trajectory characteristics, our findings align closely with previous longitudinal studies. Specifically, individuals in the low-level declining group were older and exhibited a higher baseline chronic disease burden, indicated by an increased prevalence of multimorbidity, consistent with evidence linking advanced age and accumulated health deficits with less favorable IC patterns [[29], [30], [31]]. Additionally, regression analyses revealed consistent associations between SES, CLA, and IC trajectory membership, highlighting the significance of social resources and behavioral engagement in influencing long-term IC development [[32], [33], [34], [35]]. Comparisons with other trajectory-based studies suggest that the patterns identified in this analysis correspond to commonly reported subtypes—often described as stable, declining, or improving—across various populations and analytical approaches [20,29,[36], [37], [38]]. Although some studies have focused on domain-specific trajectories, the current analysis examined overall standardized IC; both approaches converge in demonstrating that IC adhered to varied rather than uniform aging pathways.

Higher SES was consistently associated with more favorable IC trajectory membership. Utilizing the moderate-to-high stable group as the reference, individuals with higher SES exhibited lower odds of belonging to low-level declining, moderate-level declining, and low-level improving groups. These findings indicate that socioeconomic advantage correlates with the maintenance of higher IC levels over time. Notably, the interaction between SES and residential context (urban versus rural) was not statistically significant, indicating that the association between SES and IC trajectories was generally consistent across residential settings within this sample.

The observed SES gradients in IC trajectories align with evidence from long-term cohort studies conducted in Western and Chinese populations. The Whitehall II study revealed that socioeconomic inequalities significantly predict transitions from multimorbidity to disability and mortality over extended follow-up, with individuals of lower SES experiencing earlier and more pronounced functional decline [39]. Analyses based on CHARLS data have similarly recorded the influence of SES on cognitive decline, functional impairment, and multimorbidity, indicating that the expression of socioeconomic inequalities may differ across demographic subgroups, including sex and place of residence [16,17,40,41]. Several mechanisms may underlie these associations. Lower SES is often associated with reduced educational attainment and health literacy, constrained access to preventive and timely healthcare, and worse living environments [18,42]. Additionally, cumulative life-course exposures—including early-life nutrition, occupational conditions, and long-term environmental stressors—may gradually diminish physiological reserve and lower baseline IC in later life [41,[43], [44], [45]]. Chronic systemic inflammation has also been proposed as a biological pathway linking socioeconomic disadvantage with cognitive and physical decline [19,46].

Previous research has predominantly concentrated on specific leisure activities and isolated cognitive outcomes, often employing cross-sectional designs. In contrast, this study positions cognitive leisure activities (CLA) within a trajectory-based framework of intrinsic capacity (IC). By identifying heterogeneous long-term IC trajectories and utilizing mediation analysis, we provide longitudinal evidence suggesting that CLA may serve as a behavioral pathway linking socioeconomic status (SES) to patterns of IC development. Beyond these structural pathways, behavioral factors may represent additional links between SES and IC trajectories. In the current mediation analysis, CLA partially mediated the relationship between SES and IC, although the indirect effect constituted a relatively small proportion of the overall association. Consistently, higher levels of CLA were associated with reduced odds of belonging to less favorable IC trajectory groups, underscoring the role of cognitive engagement in sustaining functional capacity. Previous studies have similarly reported correlations between cognitively and socially engaging activities, including reading, playing mahjong or other strategy games, internet use, and social interaction, and better cognitive performance, emotional regulation, and psychological well-being [20,[47], [48], [49]]. From a biological and sociological perspective, engagement in CLA may stimulate brain function and enhance neuroplasticity [50,51], reduce systemic inflammation linked to IC decline [52,53], and build cognitive reserve that buffers age-related functional deterioration [54,55].Although these mechanisms offer plausible explanations, the modest mediation effect indicates that CLA constitutes only one of several pathways through which SES influences IC, alongside factors such as chronic disease burden, healthcare access, psychological health, lifestyle behaviors, and social support. Accordingly, socially engaging forms of CLA may be beneficial for older adults across diverse contexts. Active social participation may enhance positive self-perception, mitigate negative attitudes toward aging, strengthen confidence in functional abilities, and alleviate the perceived burden associated with IC decline [56].

This study has several notable strengths. First, the use of longitudinal data from the nationally representative CHARLS cohort enhances the generalizability of our findings to middle-aged and older adults in China. Second, the application of group-based trajectory modeling enabled the identification of heterogeneous long-term IC trajectories rather than assuming a uniform age-related decline. Third, by integrating trajectory analysis with mediation modeling, this study provides evidence suggesting that CLA may serve as a behavioral pathway linking SES to IC trajectories. Compared with prior research that has largely focused on cross-sectional associations between specific leisure activities and single cognitive outcomes, our trajectory–mediation framework offers a more comprehensive perspective on functional aging and extends the literature beyond cognition-specific endpoints. Nevertheless, several limitations should be acknowledged. First, IC and CLA were partly based on self-reported measures, which may introduce recall or reporting bias; however, such misclassification is likely to be non-differential and may bias estimates toward the null. Second, although we adjusted for multiple sociodemographic and health-related covariates, residual confounding cannot be fully ruled out. Third, despite the longitudinal design, the observational nature of the data limits causal inference. Finally, the follow-up period (2011–2015) may not capture longer-term IC trajectories, and future studies with extended follow-up are warranted.

Collectively, findings from this study and previous trajectory-based research suggest that long-term IC trajectories are shaped by the combined effects of aging-related health burden and socioeconomic factors. Although the number and distribution of trajectory classes vary across studies—attributable to differences in sample characteristics, follow-up duration, disease profiles, and modeling strategies—the recurring pattern of stability, decline, and improvement provides a coherent framework for understanding heterogeneity in functional aging. These results support the utility of trajectory-based approaches for characterizing long-term IC development and identifying population groups at increased risk of unfavorable aging outcomes.

This study has several notable strengths. First, the use of longitudinal data from the nationally representative CHARLS cohort enhances the generalizability of our findings to middle-aged and older adults in China. Second, group-based trajectory modeling enabled the identification of heterogeneous long-term IC trajectories rather than assuming a uniform age-related decline. Third, by integrating trajectory analysis with mediation modeling, this study suggests that CLA may serve as a behavioral pathway linking SES to IC trajectories. Compared with prior research that has largely focused on cross-sectional associations between specific leisure activities and single cognitive outcomes, our trajectory–mediation framework offers a more comprehensive perspective on functional aging and extends the literature beyond cognition-specific endpoints. However, several limitations should be acknowledged. First, IC and CLA were partly based on self-reported measures, which may introduce recall or reporting bias; however, such misclassification is likely to be non-differential and may bias estimates toward the null. Second, although we adjusted for multiple sociodemographic and health-related covariates, residual confounding cannot be fully ruled out. Third, despite the longitudinal design, the observational nature of the data limits causal inference. Finally, the follow-up period (2011–2015) may not capture longer-term IC trajectories, and future studies with extended follow-up are warranted.

## 5 Conclusions

This study demonstrated that IC among Chinese adults aged ≥ 45 years follows distinct long-term trajectories, highlighting significant heterogeneity in functional aging. SES was consistently correlated with trajectory membership, with higher SES associated with increased probability of maintaining favorable IC and lower SES linked to persistently low or declining trajectories. Cognitive leisure activities partially accounted for the SES–IC association, indicating that behavioral engagement represents one modifiable pathway within broader socioeconomic influences. These findings support integrated strategies that reduce socioeconomic disparities and expand accessible opportunities for cognitive and social engagement, alongside healthcare and community support, to help preserve IC and foster more equitable aging trajectories.

## CRediT authorship contribution statement

All authors meet the authorship criteria as outlined in the Uniform Requirements for Manuscripts Submitted to Biomedical Journals, and all authors have reviewed and approved the final manuscript.

QMY: Conceptualization; Data curation; Formal analysis; Methodology; Visualization; Writing – original draft. WJL: Conceptualization; Formal analysis; Methodology; Funding acquisition; Writing – original draft. WL: Data curation; Formal analysis; Methodology; Writing – review & editing. XLY: Conceptualization; Investigation; Project administration; Writing – review & editing. HYQ: Methodology; Software; Visualization; Writing – review & editing. RLY: Conceptualization; Software; Visualization; Writing – review & editing. WXH: Conceptualization; Supervision; Project administration; Writing – review & editing.

## Ethical statement

The CHARLS study was approved by the Biomedical Ethics Review Committee of Peking University (IRB00001052-11015). Written informed consent was obtained from all participants.

## Declaration of Generative AI and AI-assisted technologies in the writing process

During the preparation of this manuscript, the authors used ChatGPT (OpenAI) to assist with language polishing, with the aim of improving readability and clarity. All content was subsequently reviewed and revised by the authors, who take full responsibility for the accuracy and integrity of the work.

## Funding

This research received no specific grant from any funding agency in the public, commercial, or not-for-profit sectors.

## Brief summary

This longitudinal study of middle-aged and older adults in China suggests that socioeconomic status is associated with distinct trajectories of intrinsic capacity. Cognitive leisure activities partially mediate this association, indicating that promoting cognitive engagement may help reduce socioeconomic differences in intrinsic capacity and support healthier ageing trajectories.

## Availability of data and materials

The datasets analyzed in this study are publicly available from the China Health and Retirement Longitudinal Study (CHARLS): http://www.charls.pku.edu.cn/.

## Declaration of competing interest

The authors declare no conflicts of interest.

## Footnotes

Appendix A Supplementary data associated with this article can be found, in the online version, at http://dx.doi.org/10.1016/j.jnha.2026.100809.
