# Supplementary material for: Socioeconomic status and intrinsic capacity trajectories among middle-aged and older adults in China: mediating role of cognitive leisure activities
Source: J Nutr Health Aging. 2026 Feb 21;30(4):100809. doi: 10.1016/j.jnha.2026.100809 (PMC12945631; doi:10.1016/j.jnha.2026.100809)
Supplement: Supplementary file 1 [file mmc1.docx]

**Supplementary Material**

**Socioeconomic Status and Intrinsic Capacity Trajectories in Chinese Middle-Aged and Older Adults: The Mediating Role of Cognitive Leisure Activities**

- **Evaluation techniques for the five domains of intrinsic capacity.**

Following a comprehensive review of the literature, the IC score for this study was established as the aggregate of the scores (ranging from 0 to 10) across the five dimensions.

1. Locomotion: This dimension was evaluated using the SPPB scale, encompassing assessments of walking speed, balance, and chair rise. Participants were scored based on their performance in a 4-meter walk: those completing it in less than 4.82 seconds received a score of 4, those taking between 4.82 and 6.20 seconds received a score of 3, those taking between 6.21 and 8.70 seconds received a score of 2, those taking more than 8.70 seconds received a score of 1, and those unable to complete the walk received a score of 0. For the balance assessment, participants who could stand with feet together and half-foot spacing for 10 seconds were assigned a score of 1, while those unable to maintain the stance for 10 seconds or who did not attempt it were assigned a score of 0.The scoring system for maintaining a full-foot spacing was as follows: a duration of 10 seconds was awarded a score of 2, a duration between 3 and 9.9 seconds received a score of 1, and a duration of less than 3 seconds or failure to attempt was assigned a score of 0. The time taken to complete five chair rises was categorized into the following intervals: less than 11.19 seconds, 11.2-13.69 seconds, 13.7-16.69 seconds, 16.7-59.9 seconds, and greater than 60 seconds, or inability to complete the task. These categories were assigned scores ranging from 4 to 0 points in descending order. The Short Physical Performance Battery (SPPB) score was calculated as the sum of these three components. The motor dimension was subsequently classified into three levels based on the SPPB score: level 0 (SPPB score between 0 and 2), level 1 (SPPB score between 3 and 9), and level 2 (SPPB score between 10 and 12).

The domain of locomotion was operationalized utilizing the following variables from the China Health and Retirement Longitudinal Study (CHARLS): gg002, gg003, qd002, qe002, qf002, qh002, and qh003.

1. Sensory: The sensory dimension is comprised of two components: vision and hearing. Within the vision component, participants responded to two items: "How would you rate your eyesight for seeing objects at a distance: excellent, very good, good, fair, or poor?" and "How would you rate your eyesight for seeing objects up close: excellent, very good, good, fair, or poor?" Participants who self-reported their eyesight as excellent, very good, or good were assigned a score of 1, those reporting fair received a score of 0.5, and those reporting poor, as well as individuals who wore glasses or were blind, were assigned a score of 0. The hearing component included the item: "How would you rate your hearing: excellent, very good, good, fair, or poor?" The scoring criteria for hearing were identical to those applied to vision.

The sensory domain was assessed using CHARLS variables da032, da033, da034, da035, da036, da037, da038, da039.

1. Vitality: The vitality dimension encompasses two key components: lung capacity and grip strength. Lung capacity was assessed using spirometry, with the maximum value from three measurements being recorded. These values were scored as 0 or 1 for males and females based on threshold values of 350 L/min and 220 L/min, respectively. Grip strength was evaluated by measuring the maximum strength of the dominant hand, with scores assigned as 0 or 1 for males and females according to threshold values of 28 kg and 18 kg, respectively.

The vitality domain was measured using the following CHARLS variables: qb002, qb003, qb004, qc003, qc004, qc005, qc006.

1. Psychology : The 10-item version of the Centre for Epidemiological Studies Depression Scale (CESD-10) is utilized to evaluate depressive symptoms. This scale comprises 10 items, each offering four response options reflecting the frequency of symptoms over the past week: little or no time (< 1 day), occasionally (1-2 days), sometimes or about half the time (3-4 days), and most or all of the time (5-7 days). The cumulative scores range from 0 to 30, with higher scores indicating more severe depressive symptoms. The psychological dimensions are categorized as follows: a score of 0 is assigned for CESD scores between 20 and 30, a score of 1 for CESD scores between 10 and 19, and a score of 2 for CESD scores between 0 and 9.

The psychology domain was captured using CHARLS variables dc009, dc010, dc011, dc012, dc013, dc014, dc015, dc016, dc017, dc018.

1. Cognition: The cognitive assessment tools employed in the China Health and Retirement Longitudinal Study (CHARLS) encompass evaluations of intelligence and situational memory. Intelligence was measured using a series of tasks, including the subtraction of 7 from 100 performed five consecutive times, identification of the current year, month, day, day of the week, and season, and an assessment of visuospatial ability through the drawing of two overlapping pentagons. The scores for these tasks range from 0 to 11. Situational memory was evaluated based on the number of immediate and delayed recalls of 10 Chinese words, with scores ranging from 0 to 10. Each of these two dimensions was assigned a score of 0 if any dimension fell below one standard deviation, while scores of 1 were given otherwise. The overall cognitive dimension score was calculated as the sum of the two dimensions, yielding a total score range of 0 to 2.

The cognition domain was assessed using CHARLS variables dc019, dc020, dc021, dc022, dc023, dc024, dc025, dc026, dc027.
